# Enhancing Internal Limiting Membrane Inverted Flap Outcomes With Amniotic Solution for Chronic and Large Macular Holes

**DOI:** 10.1097/IAE.0000000000004624

**Published:** 2025-08-01

**Authors:** Tomaso Caporossi, Giulia Grieco, Matteo Mario Carlà, Alessandra Scampoli, Lorenzo Governatori, Stanislao Rizzo

**Affiliations:** * Catholic University Sacro Cuore, Rome, Italy; and; † Vitreoretinal Surgery Unit, Fatebenefratelli Isola Tiberina Gemelli Isola Hospital, Catholic University “Sacro Cuore”, Rome, Italy.

**Keywords:** macular hole, vitrectomy, human amniotic membrane

## Abstract

**Purpose:**

This study assesses the effectiveness of a modified inverted internal limiting membrane flap technique combined with human amniotic membrane solution for treating large, chronic, and idiopathic conditions.

**Methods:**

Fourteen eyes from 14 patients underwent pars plana vitrectomy with a temporal internal limiting membrane flap technique and hAM application. Two methodologies for hAM preparation were compared: the direct injection of an hAM suspension and a refined method that used a vitrectomy probe to produce a homogeneous hAM solution. All eyes were tamponed with 20% sulfur hexafluoride.

**Results:**

Postoperative outcomes showed significant improvements in best-corrected visual acuity and successful macular hole closure in all cases. The homogeneous hAM solution yielded superior anatomical and functional outcomes compared with the initial suspension method. It also showed better retinal layer integration without any adverse effects like displaced fragments or visual disturbances.

**Conclusion:**

This study highlights hAM solution as a promising supplement in macular hole surgery, enhancing flap stabilization and retinal regeneration. Larger studies are needed to confirm its long-term efficacy and safety.

## Abstract 2

Supplemental Digital Content is Available in the Text.

The full-thickness macular hole (FTMH) is an anatomical defect in the fovea, characterized by the complete disruption of all neural retinal layers. It extends from the internal limiting membrane (ILM) to the retinal pigmented epithelium (RPE).^1^ Idiopathic macular holes occur at an annual incidence rate of approximately eight cases per 100,000 individuals. They are more prevalent in women than in men, with a ratio of 2:1.^2^ These holes are frequently observed in individuals aged between 60 and 80 years, but can also affect younger individuals, especially those with myopia.^3^

The first documented case of a macular hole dates back to 1869 when Knapp described an instance related to trauma. A deeper understanding of this condition, however, began with J.D.M. Gass. He introduced a staging system based on biomicroscopic observations, which classified the progression of macular holes from impending to full-thickness stages.^4,5^

In 2013, the International Vitreomacular Traction Study (IVTS) Group developed an optical coherence tomography (OCT)-based anatomical classification system for vitreomacular interface diseases, which includes macular holes. This system classifies FTMHs by size into small (aperture size less than 250 *µ*m), medium (250–400 *µ*m), and large (more than 400 *µ*m), further distinguishing cases by the presence or absence of vitreous attachment.^1^ More recently, in 2023, the CLOSE study group proposed an updated surgical classification for large FTMHs. This new system aims to enhance surgical decision making by correlating hole size with the most effective treatment strategies.^6^

If left untreated, idiopathic macular holes typically increase in size and severity, leading to progressive central vision loss. Pars plana vitrectomy has long been the standard treatment, offering high success rates. This procedure was first successfully performed in 1991 by Kelly and Wendel,^7^ who developed a method involving extensive vitrectomy, detachment of the posterior vitreous cortex, removal of any epiretinal membrane, comprehensive fluid–gas exchange, and postoperative face-down positioning. ILM peeling as a technique to enhance macular hole closure rates was first described by Eckardt et al.^8^ in 1997. The procedure has since evolved, and the use of dyes has greatly facilitated the visualization of the ILM. End-gripping forceps are used to pinch and lift a flap of the ILM without disturbing the optic nerve fibres. The ILM is peeled off in a circular motion, creating a “maculorrhexis.” Michalewska et al.^9^ proposed retaining the dissected ILM within the eye to cover the hole with an ILM flap, particularly in myopic eyes and large or refractory macular holes. Many authors have found that this technique yields better results in recovery of visual acuity and closure of the hole compared with peeling alone.^10,11^

The human amniotic membrane (hAM), which is the innermost, semitransparent, avascular layer of the placenta, exhibits excellent antiangiogenic and antimicrobial properties with low immunogenicity. The hAM secretes various cytokines, such as TGF-α, TGF-β, fibroblast growth factors, epithelial growth factor, and hepatocyte growth factor. In addition, it aids in nerve reconstruction in animal models and serves as a reservoir for neurotrophic factors like NGF, BDNF, NT-3, GDNF, and CNTF, promoting cell proliferation and neuroprotection.^12^ It has been widely used in ocular surface reconstruction. In vitreoretinal applications, hAM supports RPE cell growth and secretes essential growth factors for retinal health. Rizzo and colleagues^13^ pioneered its use for repairing refractory FTMHs and retinal breaks. In such instances, hAM serves as a plug, promotes retinal regeneration, stimulates gliosis, and aids in retinal layer differentiation and migration.

The hAM graft can be sourced as cryopreserved, dehydrated, or lyophilized, with cryopreserved hAM being the most common and beneficial.^14,15^ The patch is typically cut with a punch of varying diameters, most commonly 1 to 2 mm, and is adjusted using vitreoretinal scissors to slightly exceed the internal diameter of the FTMH, by approximately 300 to 500 *µ*m larger than the hole itself.^16^ Once inside the vitreous chamber, the hAM is manipulated under fluid or perfluorocarbon liquid, ensuring the chorion layer faces the RPE. The patch is then gently inserted under the edges of the FTMH to completely cover the hole. After a fluid–air exchange, the eye is filled with either air or SF6 gas. Postoperatively, patients are advised to maintain a face-down position.

For refractory FTMHs, including those with high myopia, hAM transplantation achieves closure rates of 57.1% to 100%, with significant best-corrected visual acuity (BCVA) improvement and OCT-detected graft integration lasting up to 13 months.^17,18^ Rizzo et al.^13^ highlighted the differentiation of external retinal layers and the centripetal migration of hole edges, with external limiting membrane (ELM) and ellipsoid zone (EZ) regeneration correlating with better visual acuity and foveal sensitivity improvements, peaking at 6 months. Reconstructed capillary plexuses around the parafoveal region also support visual gains, particularly in nonmyopic eyes. However, some cases show persistent disorganization of ELM and EZ.

## Materials and Methods

### Population and Study Design

The investigation constituted a prospective, controlled, interventional, single-center study, involving a consecutive series of 14 eyes from 14 patients afflicted with idiopathic, chronic, and large FTMH. This study was conducted within the Department of Vitreoretinal Surgery at Ospedale Isola Tiberina—Gemelli Isola, Rome, Italy, between January and December 2024.

The inclusion criteria were age 18 or older, preoperative pseudophakia, a clinical and instrumental diagnosis of chronic^19^ (symptoms of central vision loss lasting more than 1 year), a large (>400 *µ*m), and idiopathic FTMH. Exclusion criteria included any previous ocular surgery, barring uneventful cataract extraction, and any concurrent ocular or systemic diseases that could potentially affect BCVA.

All patients underwent a comprehensive ophthalmic evaluation, which included the best-corrected LogMAR visual acuity measurement, a dilated anterior and posterior segment examination, concluded with OCT volumetric scans of the macula (Optovue AvantiRTVue XR, software version 6.1.0.4; Optovue, Inc., Fremont, CA). Baseline characteristics are summarized in Table 1.

**Table 1.** Baseline demographic and clinical characteristics of patients in Group 1 and Group 2

Baseline Characteristics	Group 1	Group 2	*P*
Age (years), mean ± SD	55.5 ± 27.5	68.8 ± 28.9	0.313
Sex, n (%)			
Female	1 (25)	4 (40)	
Male	3 (75)	6 (60)	
VA, logMAR, mean ± SD	1.35 ± 0.4	1.2 ± 0.4	0.375
Basal macular hole diameter, mean ± SD	620 ± 183	704 ± 197	0.322

logMAR, logarithm of the minimum angle of resolution; VA, visual acuity.

The preparation of Amniotic Membrane Extract Eye Drops (AMEED) begins with the collection of placentas from donors undergoing elective caesarean deliveries after a minimum of 35 weeks of gestation. Placentas are processed shortly after collection. The amniotic membrane (AM) is then cut into fragments and ground in a sterile balanced salt solution (1 g wet weight/10 mL BSS). The AM suspension is divided into aliquots and stored at −80°C until it is delivered to the patient. The Mestre Eye Bank is responsible for the delivery of all AMEED (see Protocol for Preparing the AMEED, **Supplemental Digital Content 1**, http://links.lww.com/IAE/C689).

For all patients, follow-up visits, which included BCVA evaluation and OCT scans, were conducted during the second postoperative week and the first, third, and sixth postoperative months. Throughout the follow-up period, no additional ophthalmologic treatments were administered to any patient.

All clinical procedures were conducted following the principles of the Declaration of Helsinki. Written informed consent was obtained from all patients before their enrollment.

### Surgical Technique

All interventions were performed by a single experienced vitreoretinal surgeon (T.C), after peribulbar anesthesia and antisepsis with a povidone-iodine solution. A standard 25-gauge three-port vitrectomy at 7500 cpm cut rate (Constellation Vision System, Alcon Laboratories Inc, Fort Worth, TX) with accurate vitreous base shaving was executed. A mixture of vital dyes (Dual Blue; DORC International, Zuidlan, Netherlands) was injected into the vitreous chamber and roughly washed out with active aspiration after 30 seconds; the ILM was peeled to create a temporal inverted flap covering the FTMH. After the peeling, a complete fluid/air exchange was performed, with careful attention to the final disposition of the ILM flap over the macular hole. The hAM suspension were then applied as follows: in the first four patients of the study, the hAM suspension was directly injected over the macular hole through a backflush needle connected to the syringe containing the hAM suspension. In the subsequent 10 cases, the hAM suspension was aspirated with the vitrectome probe with an activated cut. Proportional reflux was then selected, the tip of the vitrectome was moved close to the surface of the macula, and by gently pressing the footswitch of the vitrectome, the hAM solution was released over the macular hole with a steady flow. This method allows for a more uniform solution of hAM particles instead of a suspension: in fact all the amniotic membrane suspensions used have the same origin but we observed that the use of the vitrectome allows us to reduce the size of the hAM fragments, making them no longer visible.

Both groups were tamponed with 20% sulfur hexafluoride (SF_6_). A face-up position was maintained for the first hour immediately after surgery, and a face-down position was maintained for the first five postoperative days. The step-by-step surgical technique is demonstrated in **Supplemental Digital Content 1** (see **Video**, http://links.lww.com/IAE/C690).

### Statistical Analysis

Statistical analysis was performed using STATA software, version 15.1 (StataCorp, College Station, TX). Descriptive statistics were used to summarize the mean values and standard deviations of all numerical data. The Pearson coefficient was used as a method to examine the correlation between variables. A *P*-value of less than 0.05 was deemed statistically significant.

## Results

The study was conducted using 25-G pars plana vitrectomy, performed by one surgeon (T.C.) on 14 eyes (eight women and six men; mean age: 65 years; age range: 18–86 years; median axial length 24.05; axial length range: 23.11–30.01 mm). The mean preoperative BCVA was 23 ± 21 ETDRS letters (1.24 ± 0.42 logMAR; Snellen 20/350), and the final postoperative BCVA improved to 50 ± 26 ETDRS letters (0.71 ± 0.51 logMAR; Snellen 20/100), representing a statistically significant improvement from baseline (*P* < 0.02).

In the initial four patients treated without the shredded solution, the mean preoperative BCVA was 18 ± 24.5 ETDRS letters (1.35 ± 0.49 logMAR; Snellen 20/450), and the postoperative BCVA reached 25 ± 35 ETDRS letters (1.20 ± 0.70 logMAR; Snellen 20/320) (*P* < 0.25). In the subsequent 10 eyes treated with the shredded solution, the mean preoperative BCVA was 25 ± 22 ETDRS letters (1.20 ± 0.44 logMAR; Snellen 20/320), and the final postoperative BCVA improved to 59 ± 16 ETDRS letters (0.52 ± 0.32 logMAR; Snellen 20/65) (*P* < 0.03).

**Fig. 1.**
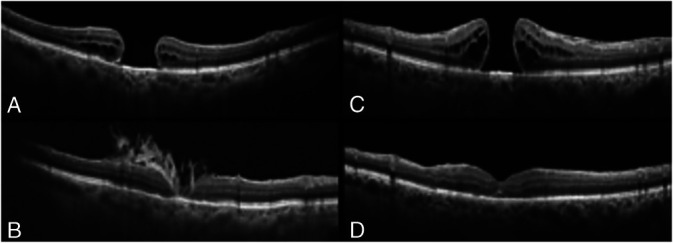
Preoperative structural OCT (**A**). Six-month postoperative OCT shows the macular hole closure with the presence of amniotic tissues inside the macular hole. The closure pattern is a 2A where interposed filling tissue plugs the foveolar dehiscence throughout all layers (**B**). Preoperative OCT (**C**). Six-month postoperative OCT does not show the presence of the HAM tissue (**D**).

**Fig. 2.**
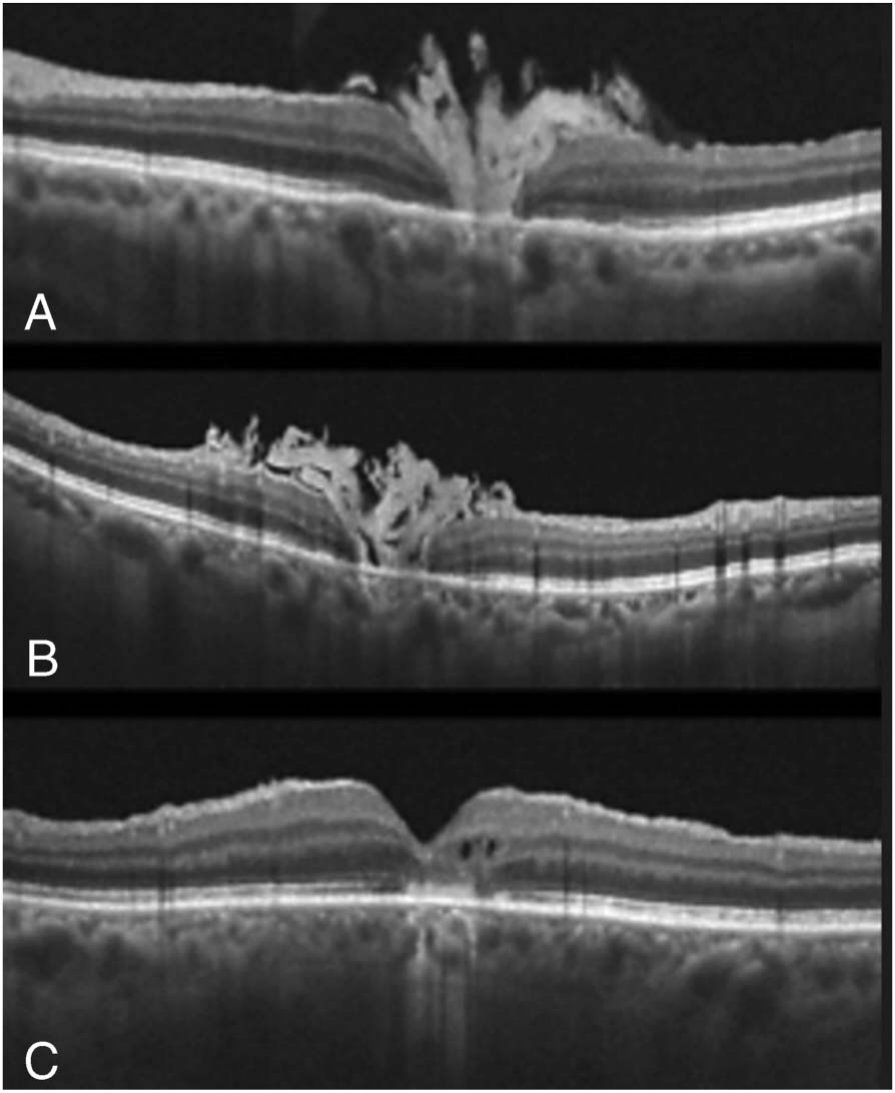
Macular hole closure patterns in the first group: 2A with interposed filling tissue which plugs the retina hesitating a foveolar dehiscence throughout all the retinal layers (**A** and **B**); 2B with reconstruction of the inner foveal layer, the external foveal layers were interrupted by the presence of the HMA material (**C**).

**Fig. 3.**
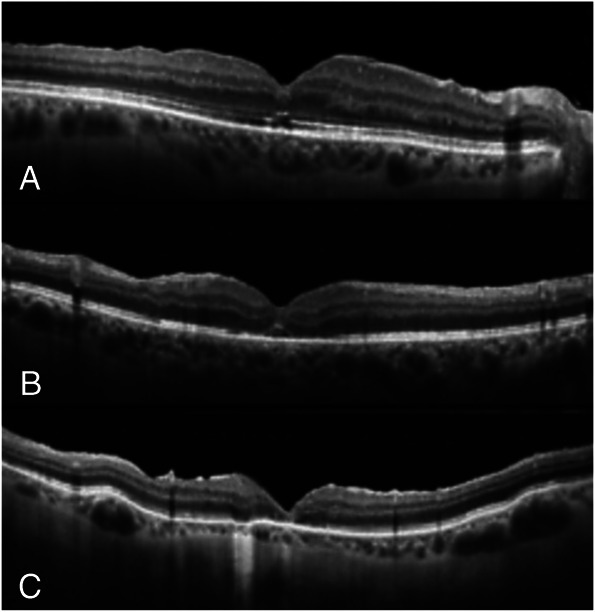
Macular hole closure patterns in the second group: 1A with complete reconstructions of all the foveal layers (**A**); 1B where is detectable an interruption of the external foveal layers (**B**); 1C with an interruption of the internal retinal layers (**C**).

The average basal FTMH diameter was 680 ± 270 *µ*m, and all the holes were successfully closed (Figure 1). Particles of the AM from the directly applied hAM suspension altered the final integrity of the retinal layers in the first four patients treated. The closure pattern of the macular holes in these initial four patients aligned with the Type 2 classification in the closure pattern reclassification proposed by Rossi et al.^20^ Specifically, three eyes exhibited a Type 2A pattern, with tissue filling and interposing the foveolar dehiscence across all layers, and one eye displayed a Type 2B pattern, demonstrating the reconstitution of normal inner retinal layers (Figure 2). Such results could be conjectured as the main reason for the underwhelming functional recovery. Consequently, we opted to prepare the solution using a vitrectome probe to achieve a more consistent fluid. The postsurgical images reveal the integration of the hAM solution with the retinal tissue, resulting in improved postoperative anatomical and functional outcomes. Remarkably, the hAM solution was undetectable in 6-month postoperative OCT, and the closure pattern of the macular hole mirrored a Type 1A in six instances with the restoration of banded anatomy across all retinal layers, a Type 1B in two instances with a residual interruption of the external layers, and a Type 1C in two instances with an interruption of the internal layers (Figure 3).

## Discussion

The inverted flap technique poses a challenge in guaranteeing the stability of the ILM flap over the macular hole during fluid–gas exchange. To address this issue, various modifications have been proposed.

In 2014, Shin et al.^21^ used perfluoro-n-octane to stabilize the inverted flap. However, this method could potentially allow perfluoro-n-octane liquids to enter the subretinal space. Extracting these liquids can pose quite a challenge and may even lead to anatomical disruption. In addition, it could cause the ILM flap to roll up, thereby making its even placement over the hole difficult.

In Aurora et al.’s case report,^22^ an innovative approach diverging from the traditional circular inverted flap method was used: three separate flaps were created and anchored at the edge of the macular hole, then inverted over it like cabbage leaves. This technique provides two main advantages: first, the interlocking structure of one flap's retinal surface with another's vitreous surface enhances adhesion, decreasing the chances of flap displacement during fluid exchange; second, the multilayered coverage assures a more thorough seal of the hole, forming a closed cavity between the neurosensory retina, RPE, and ILM. This may encourage the centripetal migration of tissue and aid in macular hole closure. A variant of this technique was suggested by Habib et al.^23^: using ILM forceps, multiple semilunar flaps were developed starting from the nasal side toward the macular hole, bypassing the papillomacular bundle. Traction was applied either temporally or vertically, with the optic disc acting as an anchor to prevent putting excessive strain on the detached retina, mitigating the risk of retinal folding or additional breaks. Each flap was pulled from the edge of the preceding one to minimize tissue damage while maintaining a hinge that linked the flap's base to the macular hole. All flaps were then arranged over the macular hole in a flower-petal configuration. This method allowed for more controlled flap creation and provided backup coverage in the event any flap was torn or flipped back.

Romano et al.^24^ explore the use of cohesive viscoelastic devices (OVD) in ILM flap techniques for macular hole surgery. The study specifically focuses on two approaches: the inverted cover technique and the free flap technique. The inverted cover method was applied to a patient who had a large secondary myopic macular hole measuring 700 *µ*m at its smallest diameter. Postsurgery, the macular hole closed within 7 days and considerable anatomical restoration, including the reformation of retinal layers and foveal depression, was observed by the 14th day. The patient's visual acuity improved significantly, demonstrating the success of this method in addressing challenging macular holes.

The authors attribute these outcomes to the stabilizing properties of OVD. It maintains flap positioning during surgical maneuvers and provides a supportive scaffold for cellular repair and glial proliferation. Unlike perfluorocarbon liquids, which require careful removal because of the risks of toxicity, OVD dissolves naturally. This reduces the potential for complications and simplifies postoperative care.

The study also contrasts the cover and fill techniques for ILM flaps, noting that the cover method is connected with superior visual outcomes and less glial proliferation, leading to a better recovery of the outer retinal layer. Imaging results in the cover group showcase the reestablishment of the foveal contour and the alignment of photoreceptors, both critical for visual function.

The use of OVDs for stabilizing inverted flaps in macular holes has also been successfully explored by other authors. In 2016, Andrew delved into this with the “viscoelastic cap” method.^25^ Fan examined the “viscoelastic agent pool” technique,^26^ and Liu explored the DiscCoVisc approach (an alternative surgical method for macular hole repair with minimal posterior pole vitrectomy, devoid of fluid–air exchange, gas tamponade, and prone positioning).^27^

In 2024, Amaral and colleagues examined the role of autologous platelet concentrate (APC) as an adjunct in macular hole repair, especially for complex cases where standard surgical methods alone may be insufficient. The study analyzed six research papers (involving over 600 patients) to evaluate the efficacy and safety of APC. The authors found that APC improves anatomical closure rates compared with conventional surgical techniques. This improvement is attributed to its bioactive components, such as growth factors that stimulate cellular migration, proliferation, and tissue repair, thereby aiding the closure of the macular hole. However, the study noted that APC's impact on functional recovery, particularly improvements in BCVA, was less consistent and did not display a significant advantage over traditional methods. Concerning safety, APC usage was not associated with an increased risk of adverse outcomes or complications. The authors emphasize that the lack of standardization in APC preparation protocols could contribute to variations in results across studies.^28^

Our technique aims to address the issue of flap stability by using human AM solution instead of APC. It has been observed that APC does not lead to significant improvements in BCVA.

In all patients treated using this technique, the macular hole closed successfully, with no reported cases of infectious or inflammatory complications, increased intraocular pression (IOP), retinal detachment, or macular hole recurrence. However, our study's two approaches do provoke some considerations.

The first four patients underwent PPV with the inverted flap technique, and hAM suspension was injected directly. This group demonstrated particles of the AM that disrupted the final integrity of the retinal layers, consequently reducing the functional results. We determined that the AMEED were a suspension of AM fragments rather than a solution, resulting in a deposit of these fragments inside the hole. Furthermore, in some instances, biomicroscopic examinations revealed displaced fragments of hAM in the mid-peripheral retina, which patients reported visually detecting.

Consequently, we can deduce that fragmenting the hAM residues using a vitrectomy probe before injection was necessary for attaining a uniform solution of the amnion. Patients who were treated with this novel processing method (10 eyes) observed a significant improvement in BCVA, in addition to a complete closure of the macular hole and good reintegration of the retinal layers as per OCT (Figure 2). Furthermore, no particles or fragments were detected in the hole in OCT images or during the biomicroscopic examination.

We can, therefore, conclude that the inverted flap is essential for the proper closure of macular holes. Its stabilization could be facilitated by the suspension or the application of the hAM solution. This method has never been used before in treating idiopathic macular holes. In fact, hAM features anti-inflammatory, antifibrotic, antimicrobial, and antiangiogenic properties, along with low immunogenicity. It serves not only as a seal for the hole but also fosters retinal regeneration through its various growth factors. This encourages intraretinal gliosis and aids in the differentiation and migration of the outer retinal layers.

In conclusion, we propose a modified ILM flap technique designed to enhance the stability of the ILM flap within the macular hole, to increase the consistency and success rate of the procedure. Our findings suggest that our procedure demonstrates promising closure rates for large and chronic macular holes, accompanied by significant improvements in vision, particularly in solution form.

Further studies, with larger sample sizes and extended follow-up periods, will be required to assess the long-term efficacy of this approach in managing macular holes.

## Supplementary Material
