# Supplementary material for: Enhancing Internal Limiting Membrane Inverted Flap Outcomes With Amniotic Solution for Chronic and Large Macular Holes
Source: Retina. 2025 Aug 1;45(12):2445–51. doi: 10.1097/IAE.0000000000004624 (PMC12637129; doi:10.1097/IAE.0000000000004624)
Supplement: Supplementary file 1 [file retina-45-2445-s001.docx]

**Protocol for Preparing the AMEED**

Here is the protocol provided by the Mestre Eye Bank for the preparation of the amniotic membrane suspension (AMEED), used in the treatment of ocular surface diseases. To avoid redundancy and excessive length, we have decided to include this section as a supplementary addition for those interested in the technique.

AMEED (amniotic membrane extract eye drops) preparation started with the collection of placentas from donors undergoing elective caesarean delivery after a minimum of 35 weeks of gestation, in compliance with established national regulation (<http://www.trapianti.salute.gov.it/>). Contraindication include malignant pathologies, fetal malformation or pathology, plus serologic and molecular screening for transmissible diseases. The placentas were processed shortly after retrieval. The AM was carefully detached from the underlying chorion and rinsed with 0.9% NaCl to remove residual blood and debris. AM was subsequently immersed in antibiotic cocktail solution made of vancomycin 100 𝜇g/ml (Hospira, Naples, Italy), meropenem 200 𝜇g/ml (Fresenius Kabi, Verona, Italy), and gentamicin 200 𝜇g/ml (Fisiopharma, Salerno, Italy), dissolved in BASE medium (Alchimia, Pordenone, Italy) overnight at +4°C of temperature. Hence, AM was cut in fragments and ground in sterile balanced salt solution (1 g wet weight/10 ml BSS). This procedure does not represent a substantial manipulation (Annex I of Regulation EC (No). 1394/2007), thereby preserving the AM biological characteristics and the legal status of a tissue processed and distributed according to the EU/national regulation for tissue banking and transplantation (Guide to quality and safety of tissues and cells from human application. 2019, European Directorate for the Quality of Medicines & HealthCare of the Council of Europe (EDQM). ISBN 978-92-871-8945-5). AM eyedrop preparation was aliquoted and stored at -80°C until delivery to the patient.
